# Supplementary material for: Development of an MRI-Guided Approach to Selective Internal Radiation Therapy Using Holmium-166 Microspheres
Source: Cancers (Basel). 2021 Oct 30;13(21):5462. doi: 10.3390/cancers13215462 (PMC8582586; doi:10.3390/cancers13215462)
Supplement: Supplementary file 1 [file cancers-13-05462-s001.zip › 20210804 Supplementary data.pdf]

Supplementary data

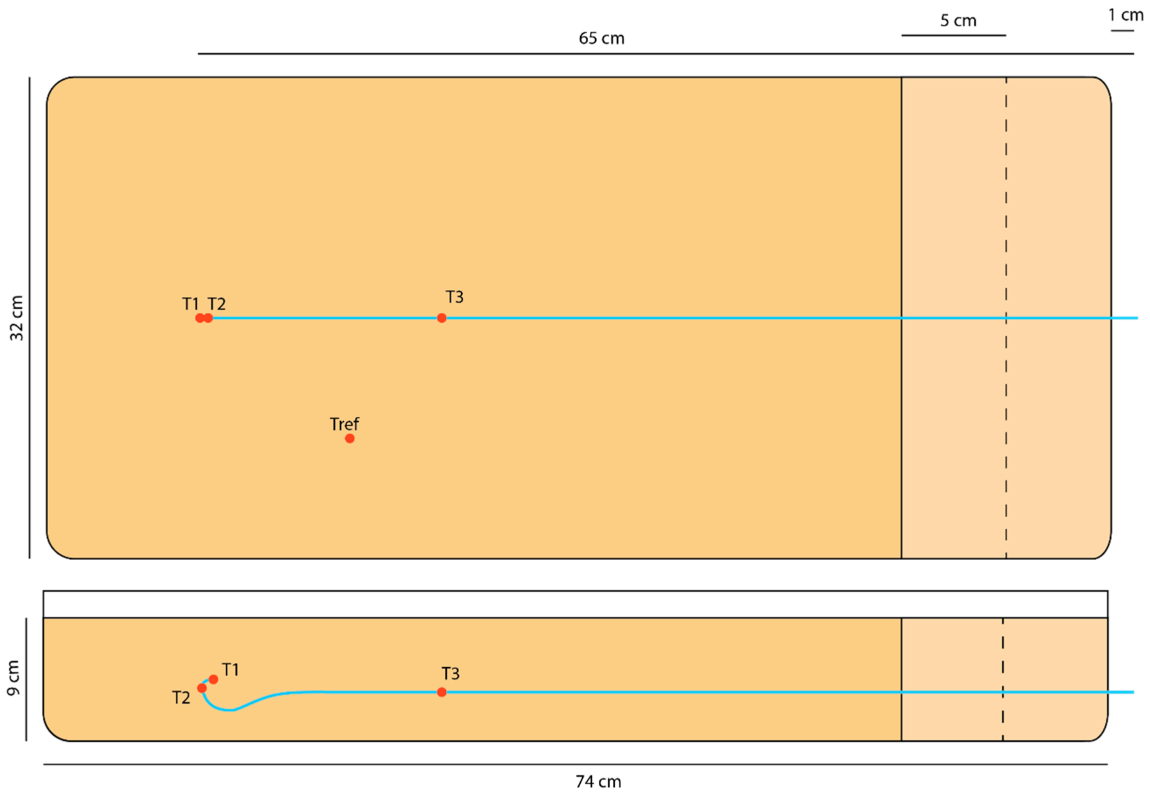

Figure S1: Design of the agarose phantom in which the radiofrequency-induced heating of catheter B was investigated. After all initial measurements, a 5 cm wide block of agarose was removed twice near the proximal end of the catheter, after which all measurements were repeated.

Table S1: Summarized results of radiofrequency-induced heating experiments performed on catheter A. Total range of maximum temperature differences is presented within brackets. As explained in figure 1, a block of 5 cm of agarose was removed at the proximal end of the phantom twice, to simulate different catheter insertion depths.

	**Insertion depth: 70 cm**			**Insertion depth: 65 cm**			**Insertion depth: 60 cm**		
**Sequence**	**T1**	**T2**	**T3**	**T1**	**T2**	**T3**	**T1**	**T2**	**T3**
T1_vibe	0,00 (0,0 - 0,0)	0,03 (0,0 - 0,1)	0,00 (-0,1 - 0,1)	-0,20 (-0,4 - 0,0)	0,03 (-0,1 - 0,1)	0,00 (0,0 - 0,0)	0,03 (-0,2 - 0,2)	0,03 (-0,2 - 0,3)	0,03 (-0,2 - 0,3)
T2_tse	0,03 (-0,1 - 0,1)	0,03 (-0,1 - 0,2)	0,00 (-0,1 - 0,1)	0,00 (-0,1 - 0,1)	0,03 (0,0 - 0,1)	0,00 (0,0 - 0,0)	-0,07 (0,0 - 0,2)	0,00 (-0,1 - 0,1)	-0,03 (-0,2 - 0,1)
T2_haste	0,03 (0,0 - 0,1)	0,17 (0,1 - 0,2)	0,00 (-0,1 - 0,1)	0,03 (0,0 - 0,1)	0,00 (0,0 - 0,0)	0,00 (0,0 - 0,0)	-0,03 (-0,1 - 0,1)	0,00 (-0,3 - 0,2)	-0,03 (-0,1 - 0,1)
TRUFI	0,00 (0,0 - 0,0)	0,00 (0,0 - 0,0)	0,00 (-0,1 - 0,1)	0,03 (-0,1 - 0,1)	0,03 (0,0 - 0,1)	0,03 (0,0 - 0,1)	0,00 (0,0 - 0,0)	0,17 (0,1 - 0,2)	0,00 (0,0 - 0,0)
TRUFI RF_{max}	0,03 (-0,1 - 0,1)	0,10 (0,0 - 0,2)	0,03 (0,0 - 0,1)	0,07 (0,0 - 0,1)	0,07 (0,0 - 0,1)	0,03 (0,0 - 0,1)	-0,03 (-0,1 - 0,0)	0,00 (-0,1 - 0,1)	-0,03 (-0,1 - 0,1)

Table S2: Summarized results of radiofrequency-induced heating experiments performed on catheter B. Total range of maximum temperature differences is presented within brackets. As explained in figure 1, a block of 5 cm of agarose was removed at the proximal end of the phantom twice, to simulate different catheter insertion depths.

	**Insertion depth: 65 cm**			**Insertion depth: 60 cm**			**Insertion depth: 55 cm**		
**Sequence**	**T1**	**T2**	**T3**	**T1**	**T2**	**T3**	**T1**	**T2**	**T3**
T1_vibe	-0,03 (-0,1 - 0,0)	0,03 (-0,1 - 0,1)	-0,03 (-0,1 - 0,0)	0,03 (0,0 - 0,1)	0,13 (0,0 - 0,2)	0,03 (0,0 - 0,1)	-0,07 (-0,2 - 0,2)	-0,07 (-0,2 - 0,2)	-0,10 (-0,2 - 0,3)
T2_tse	0,00 (-0,1 - 0,1)	-0,07 (0,0 - 0,2)	0,03 (0,0 - 0,1)	0,03 (0,0 - 0,1)	0,00 (-0,1 - 0,1)	-0,03 (-0,2 - 0,1)	0,03 (-0,2 - 0,3)	0,00 (-0,2 - 0,2)	0,07 (-0,1 - 0,3)
T2_haste	-0,03 (-0,1 - 0,0)	0,07 (-0,1 - 0,3)	-0,03 (-0,1 - 0,0)	-0,03 (-0,1 - 0,0)	0,03 (0,0 - 0,1)	-0,03 (-0,1 - 0,0)	-0,03 (-0,1 - 0,0)	-0,03 (-0,1 - 0,1)	-0,03 (-0,1 - 0,0)
TRUFI	0,00 (0,0 - 0,0)	0,03 (0,0 - 0,1)	0,03 (-0,1 - 0,1)	-0,07 (-0,1 - 0,0)	-0,07 (-0,1 - 0,1)	-0,03 (-0,1 - 0,0)	-0,03 (-0,1 - 0,1)	-0,07 (-0,1 - 0,0)	-0,03 (-0,1 - 0,1)
TRUFI RF_{max}	-0,03 (0,2 - 0,1)	-0,10 (-0,2 - 0,0)	-0,03 (-0,1 - 0,1)	0,03 (-0,1 - 0,1)	0,00 (0,0 - 0,0)	0,00 (-0,1 - 0,1)	0,00 (0,0 - 0,0)	-0,03 (-0,1 - 0,1)	-0,03 (-0,1 - 0,0)

Table S3: Summarized results of radiofrequency-induced heating experiments performed on the microcatheter (T1 and T2, see figure 1B) and catheter A (T3) in a more extreme orientation (see figure S2). Total range of maximum temperature differences is presented within brackets. As explained in figure 1, a block of 5 cm of agarose was removed at the proximal end of the phantom twice, to simulate different catheter insertion depths.

	**Insertion depth: 80 cm**			**Insertion depth: 75 cm**			**Insertion depth: 70 cm**		
**Sequence**	**T1**	**T2**	**T3**	**T1**	**T2**	**T3**	**T1**	**T2**	**T3**
T1_vibe	0,00 (0,0 - 0,0)	0,00 (0,0 - 0,0)	0,03 (0,0 - 0,1)	0,00 (-0,1 - 0,1)	0,00 (-0,1 - 0,1)	0,00 (-0,1 - 0,1)	0,00 (0,0 - 0,0)	0,00 (0,0 - 0,0)	0,00 (-0,3 - 0,2)
T2_tse	0,03 (-0,1 - 0,1)	-0,03 (-0,1 - 0,0)	0,17 (0,0 - 0,3)	0,03 (0,0 - 0,1)	0,00 (0,0 - 0,0)	0,13 (0,1 - 0,2)	-0,03 (-0,1 - 0,0)	-0,03 (-0,1 - 0,0)	0,13 (0,0 - 0,3)
T2_haste	-0,07 (-0,1 - 0,1)	-0,07 (-0,1 - 0,0)	-0,03 (-0,1 - 0,1)	0,07 (0,0 - 0,2)	-0,03 (0,0 - 0,1)	0,13 (0,1 - 0,2)	0,00 (0,0 - 0,0)	0,03 (-0,1 - 0,1)	0,10 (0,0 - 0,2)
TRUFI	0,07 (0,0 - 0,1)	0,03 (0,0 - 0,1)	**0,37 (0,3 - 0,5)**	0,07 (0,0 - 0,1)	-0,03 (-0,1 - 0,1)	0,27 (0,2 - 0,4)	0,07 (0,0 - 0,1)	0,07 (0,0 - 0,1)	**0,40 (0,4 - 0,4)**
TRUFI RF_{max}	0,03 (0,0 - 0,1)	0,03 (0,0 - 0,1)	**0,33 (0,3 - 0,4)**	0,13 (0,0 - 0,3)	0,03 (0,0 - 0,1)	**0,67 (0,6 - 0,7)**	0,07 (0,0 - 0,1)	0,07 (-0,1 - 0,1)	**0,50 (0,5 - 0,5)**
